# Association of Multidrug Resistance Gene-1 (*MDR1 C1236T*) Polymorphism with the Risk of Acute Myeloid Leukemia in a Moroccan Population

**DOI:** 10.31557/APJCP.2020.21.7.1899

**Published:** 2020-07

**Authors:** Oum Kaltoum Ait Boujmia, Sellama Nadifi, Hind Dehbi, Mouna Lamchahab, Asma Quessar

**Affiliations:** 1 *Laboratory of Cellular and Molecular Pathology, Faculty of Medicine and Pharmacy of Casablanca, University Hassan II, Casablanca, Moroccoa.*; 2 *Morocco Laboratory of medical Genetics, CHU Ibn Rochd, Casablanca, Morocco. *; 3 *Department of Onco-Hematology, Ibn Rochd University Hospital, Casablanca, Morocco. *

**Keywords:** P-gp- MDR1, acute myeloid leukemia, single nucleotide polymorphism, C1236T, Meta-analysis

## Abstract

The human multidrug resistance *MDR1* gene plays a crucial role in the absorption, transport, metabolism and elimination of harmful compounds. An impaired metabolism of these compounds related to genetic polymorphism may cause cancer such as acute myeloid leukemia AML.

**Objective:**

The present study investigated the relationship between *C1236T *polymorphism and the risk of AML development in a sample of Moroccan population.

**Methods:**

The present case-control study included 131 AML patients and 136 healthy controls. The *MDR1 C1236T* polymorphism was identified by PCR-RFLP method. Meta-analysis was performed to discuss our results. Statistical analyses were performed using SPSS, MetaGenyo and MedCalc.

**Results:**

A positive association was found between the *1236TT *mutant genotype and the risk of AML (OR 2.39; 95% CI 1.02-5.57, p= 0.04) compared to the wild type *1236CC*. In addition, the recessive model revealed that carriers of 1236TT mutant genotype were more exposed to develop AML when compared to the combined *1236CC/CT* genotype (OR: 2.27, CI: 1.01–5.05, p=0.04). The clinical parameters of AML showed no significant association. Meta-analysis demonstrated no statistically significant association between this polymorphism and AML susceptibility.

**Conclusion:**

Our study suggests that the *MDR1C1236T* polymorphism appears to be associated with the risk of AML. Further studies, including a large sample size, are needed to confirm these findings.

## Introduction

Acute myeloid leukemia (AML) is the most deadly type of leukemia (GB, 2016). As other types of cancer, the etiology of AML remains unknown. However, many risk factors were incriminated such as exposition to pesticides, radiotherapy, chemotherapy, benzene and radiation (Zeeb and Blettne, 1998; Smith et al., 2011). In addition, many genes have been reported to be associated with higher risk of AML.

*MDR1* or *ABCB1* gene is expressed in organs implicated in xenobiotics metabolism and excretion (the gastrointestinal system, kidney and liver). Therefore, *MDR1* plays a crucial role in the elimination of carcinogenic substances. The alteration of *MDR1* activity related to interindividual genetics background may lead to the development of cancers (Laura et al., 2011; Urayama et al., 2007; Ambudkay et al., 1999; Schimkel, 1997; Hattori et al., 2007).

In humans, the *MDR1* gene is located on chromosome 7, at position q21, which encodes for a transmembrane protein that plays role of ATP -dependent efflux transporter pump, called P-glycoprotein (P-gp) (Clarke et al., 2005). *MDR1* is a highly polymorphic gene. About a thousand single nucleotide polymorphisms (SNPs) have been found (Bodor et al., 2005; Hodges et al., 2011).

The synonymous *SNP C1236T* in exon12 (*rs1128503*) is one of the most studied SNPs in this gene, that leads to the alteration of *MDR1 *expression and activity (Marzolini etal., 2004). The allele frequencies of this polymorphism vary from 30% to 93% between different ethnic groups worldwide (Hodges et al., 2011) Several genetic epidemiological studies reported a positive association between this polymorphism in *MDR1* gene and cancer risk such us, acute lymphoblastic leukemia, breast cancer, acute myeloid leukemia and non-hodgkin lymphoma (Talaat et al., 2018; Abuhaliema et al., 2016; Ait Boujmia et al., 2020; Kim et al., 2014)

The purpose of the present study was to explore the relationship between *C1236T* polymorphism and AML susceptibility.

## Materials and Methods

*Patients and Methods*

*Study population*

The present case–control study included 131 AML patients and 136 healthy age-matched unrelated controls, recruited from the Department of Hematology and Pediatric Oncology, 20 August Hospital, University Hospital, Ibn Rochd, Casablanca Morocco from 2015 to 2017. Individual written informed consent was obtained from all participants. The classification of AML subtypes in patients was performed according to the criteria of the World Health Organization 2008 (WHO) (Vardiman et al., 2009).

*Genotyping of C1236T*

Genomic DNA was extracted by the salting-out protocol (Miller et al., 1998). DNA concentration was estimated by NanoVue Plus spectrophotometer. Genotypic identification of the *C1236T MDR1* polymorphism was performed using polymerase chain reaction followed by the restriction fragment length polymorphism (PCR-RFLP) technique previously described by Kassogue et al., (2014). The PCR reaction was carried out in a final volume of 25µl. The PCR products were digested overnight with HaeIII and analyzed on a 3% agarose gel stained with ethidium bromide.

*Meta-Analysis*

*Retrieval strategy*

Literature search was accomplished independently by two investigators to find all articles published before March 27, 2019 that investigating the relationship between the polymorphisms and AML risk, using the following key words: P-gp, multidrug resistance gene, *MDR1*, *ABCB1*, acute myeloid leukemia, AML, polymorphism, *C1236T*, SNP. Eligible studies were found by searching PubMed, Science Direct and Google Scholar. 

*Inclusion and exclusion criteria*

The inclusion criteria were: studies about the association between the *MDR1 C1236T* polymorphism and AML with sufficient data were included. The exclusion criteria were: chronic myeloid leukemia, acute lymphoid leukemia (CML, ALL) and lymphoma studies, review articles, studies that did not meet our study and duplicate studies.

*Pooled Studies for the Meta-Analysis*

**Figure 1:**
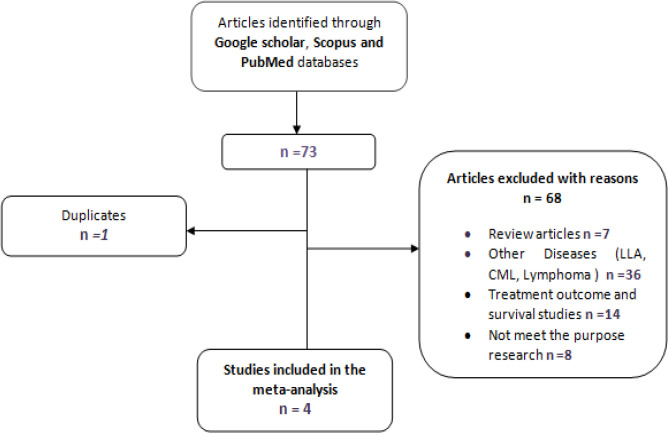
Flow Chart Explaining the Selection of the Included Studies in the Meta-Analysis

Four case-control studies investigating *MDR1 C1236T* polymorphism in AML patients were included in the present meta-analysis with 480 cases and 920 controls Figure 1.

*Statistical analysis*

Statistical analyzes were performed using SPSS (version 16; SPSS Inc., Chicago, IL, USA) and MedCalc (version 8; MedCalc Software, Mariakerke, Belgium) as well as MetaGeny (GENYO). The Hardy–Weinberg equilibrium was performed by chi-square test in patients and controls. The odds ratio (OR) with 95% confidence intervals (CI) was calculated to assess the association between the *C1236T MDR1* polymorphism and AML risk. We used either the chi-square or Fisher’s exact tests to evaluate the association between *C1236T MDR1* polymorphism and clinical parameters; a p value less than 0.05 was considered statistically significant. The dominant genetic model was defined as TT homozygote + CT heterozygote mutant genotypes compared with homozygote Wild-type genotype CC, while the recessive genetic model was defined as TT homozygote mutant compared with heterozygote+ homozygote Wild-type genotypes. For the meta-analysis the ORs were estimated using a fixed-effects model. Genetic Heterogeneity was estimated with the Cochran’s *Q* test and I^2^ statistics was used to estimate the heterogeneity among studies. A random-effects model was used to estimate common OR if heterogeneity was detected; I^2^> 50% indicated heterogeneity between studies.

## Results

In this case-control study, we examined the impact of *C1236T MDR1* gene polymorphism on the risk of developing AML in 131 patients with AML and 136 healthy controls. The genotypic distribution of* C1236T MDR1* gene polymorphism did not deviate from Hardy-Weinberg Equilibrium in controls and patients (Table 2). The clinical features of AML patients are summarized in Table 1. Mean age and sex distribution were comparable between patients and controls. When considering the FAB subtype classification, we found that 32.1% of patients had M2 and 1.1% had M7. As shown in Table 2, the genotypic frequencies were 38.9% CC, 45.8% CT, and 15.3% TT for AML patients 45.18% CC, 47.41% CT and 7.41% TT for controls, respectively. The allelic frequencies among patients were 61.83% C, 38.17%T and 68.75% C, 31.25% T in controls. In our study, the homozygous mutant genotype TT was statistically associated with the development of AML compared to the homozygous wild-type genotype CC (OR 2.39; 95% CI 1.02-5.57, p= 0.04). The same trend was observed in the recessive model where we noted that carriers of 1236TT mutant genotype were more exposed to develop AML when compared to the combined 1236CC/CT genotype (OR: 2.27, CI: 1.01–5.05, p=0.04). In contrast, the dominant model did not influence the risk of the risk of AML (P > 0.05).

Table 3 shows the distribution of clinical features of AML patients, according to the genotypes of *MDR1 C1236T* polymorphism. Our results suggest that there is no significant association between this polymorphism and age at diagnosis, sex, karyotype, FAB subtypes (P > 0.05). 

Based on the studies published on *MDR1 C1236T*, we observed no statistically significant association between this polymorphism and AML under different genetic models (P > 0.05). No heterogeneity between studies was found, I^2^ =0% Table 4.

**Table 1:** Demographic and Clinical Parameters of AML Patients and Control Subjects

	Cases (N=131)	Controls (N=136)
Sex N (%)		
Female	69 (52.7)	76(55.88)
Male	62(47.3)	60(44.12)
Sex N (%)	0.89	0.61
Age years (Mean±SD)	37.86±15.00	38.00±12.94
Range	18-74	18-75
FAB classification N (%)		
M0	2 (1.5)	
M1	27 (20.6)	
M2	42(32.1)	
M3	7(5.3)	
M4	16 (12.2)	
M5	11 (8.4)	
M6	7 (5.3)	
M7	2 (1.1)	
Other	17 (13)	
Median medullary blasts (%)	83%	
Range	(23-100)	
Median WBC (G/L)	19.7	
Range	(0.7-560.0)	
Median platelets (G/L)	35	
Range	(1-2355)	
Median hemoglobin (g/dL)	7.1	
Range	(2.50-12.80)	
Karyotype N (%)		
Normal	48 (36.6)	
Abnormal	77 (58.8)	
Missing	6 (4.6)	
Risk group		
Favorable	23 (17.6)	
Intermediate	71 (54.2)	
Adverse	31 (23.7)	
Not done	6 (4.6)	

**Table 2:** Distribution of Genotypes and Alleles of *C1236T MDR1* Gene Polymorphism in AML Patients and Controls

C1236T Genotype	Patients N (%)	Controls N (%)	OR (95% CI)	P-value
	131	136		
CC	51 (38.9)	61 (45.18)	Ref.	
CT	60 (45.8)	65 (47.41)	1.10 (0.66-1.84)	0.7
TT	20 (15.3)	10 (7.41)	2.39 (1.02-5.57)	0.04
CC/CT	111 (84.7)	126(92.59)	Ref.	
TT	20(15.3)	10 (7.41)	2.27 (1.01-5.05)	0.04
CC	51 (38.9)	61 (45.18)		
CT/TT	80 (61.1)	75(54.82)	1.27 (0.78-2.07)	0.23
Allele				
C	162 (61.83)	187(68.75)	Ref	
T	100(38.17)	85(31.25)	1.35 (0.94-1.94)	0.09
HWE: p value	0.73	0.19		

**Table 3:** Genotypic Frequencies of *C1236T MDR1* Gene Polymorphism in Patients with AML According to Clinical Parameters

	CC	CT	TT	*P*-value
Age of onset				0.16
<20	6	13	6	
>20	45	47	14	
Sex				0.73
Female	27	30	12	
Male	24	30	8	
FAB type				0.24
M0-M1	10	17	2	
M2	14	21	7	
M3	3	4	0	
M4	7	6	3	
M5	7	1	3	
M6-M7	4	5	0	
karyotype				0.95
Normal	18	22	8	
Abnormal	31	34	12	
Risk group				0.16
Favorable	11	6	6	
Intermidaite	29	33	9	
Unfavorable	9	18	4	

**Table 4:** Pooled Analysis of Studies Exploring the Relationship between *MDR1 C1236T* Polymorphism and AML Risk

	Recessive model			Dominant model			Allele contrast model		
Study	OR	95 % CI	*P*	OR	95 % CI	*P*	OR	95 % CI	*P*
AL-Faisal et al. 2014 [23].	0.81	0.16– 3.91		2.77	0.59-13.05		1.39	0.51-3.83	
Jingdong et al. 2016 [24].	0.91	0.61- 1.36		0.72	0.39-1.34		0.88	1.06- 1.83	
Feng et al. 2016 [25].	0.92	0.62- 1.37		0.89	0.49-1.60		0.93	1.13 – 1.97	
Green et al. 2012 [26].	1.12	0.66- 1.92		0.96	0.60- 1.53		1.02	0.38- 2.92	
Total (fixed effects)	0.95	0.75 to 1.22	0.73	0.91	0.67-1.24	0.57	0.95	1.04- 1.34	0.59
Total (random effects)	0.95	0.75 to 1.22	0.73	0.91	0.67-1.24	0.58	0.95	1.00- 1.39	0.59
Test for heterogeneity	Q=0.47	DF=3		Q=2.57	DF=3		Q=0.98	DF=3	
	I^2^=0%	ph=0.92		I^2^=0%	ph=0,46		I^2^=0%	ph=0.80	

**Figure 2:**
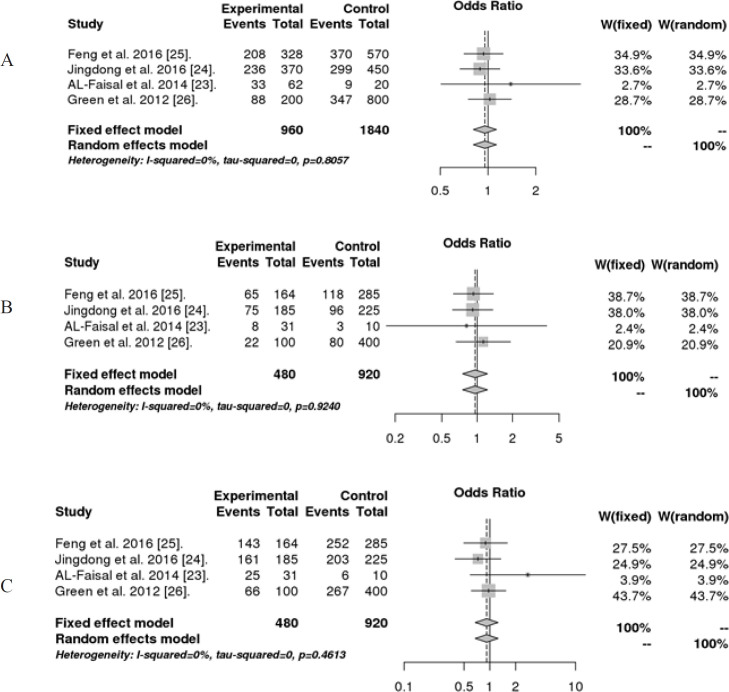
Forest Plots of Odds Ratios for the Association between *MDR1 C1236T *Polymorphism in Different Genetic Models: A, Allele contrast model; B, Recessive model; C, Dominant model

## Discussion

In this case-control study, we investigated the impact of *MDR1 C1236T* polymorphism on acute myeloid leukemia risk in a sample of Moroccan population and we found that the mutant TT genotype of *MDR1 C1236T* polymorphism was associated with the susceptibility of acute myeloid leukemia when compared to the wild type CC genotype. The most frequently observed FAB subtype in our study was M2; this result is consistent with our previous studies (Ait Boujmia et al., 2017; Ait Boujmia et al., 2020). Other studies have reported similar results (Habdous et al., 2004; Zendehdel et al., 2009). Regarding the correlation between this polymorphism and clinical parameters, we found that there was no statistically significant positive association between the *MDR1 C1236T* polymorphism and gender, age, karyotype and FAB types. Interestingly, our results showed a significant association between the TT homozygote mutant genotype and the risk of AML with an odds ratio of 2.39 (95% CI: 1.02-5.57, P=0.04). AL-Faisal et al. reported a similar result in Iraqi population (Al Faisal and Al yaqubi, 2014).

In contrast, our Meta-Analysis and some studies have not shown an influence of this polymorphism and the risk of developing AML. Jingdong et al., (2016) reported that there is no significant association between *MDR1 C1236T* gene polymorphism and AML risk. Feng et al., (2016) reported that no significant relationship between the *ABCB1 C1236T* variant and AML. Green et al., (2012) also found no significant difference in genotype frequencies between AML patients and the reference population. The difference in results between studies of *MDR1 C1236T* gene polymorphism could be explained by many factors such as the genetic background, the small sample size, difference in genotyping methods, and the mode of living and it should be noted that the frequency of this SNP varied according the ethnic group and to geographic location (Kassogue et al., 2013).

The influence of *MDR1 C1236T* gene polymorphism on the risk of AML could be explained by the intracellular accumulation of toxic and carcinogenic substances due to the low expression of *MDR1* gene. Furthermore, many studies reported that the diminished MDR1 expression leads to increase DNA damage and Cytogenetic aberrations that play a critical role in the leukemogenesis (Ait Boujmia et al., 2017; Ait Boujmia et al., 2020).

In conclusion, this study results suggested that the *MDR1 C1236T* polymorphism could influence the risk of developing adult acute myeloid leukemia. Future studies with large sample size are necessary to confirm the association between *MDR1 C1236T* polymorphism and AML risk. 

## Ethics approval / Consent Informed

The present case-control study was approved by the Ethical Committee of Hassan II University, School of Medicine and Pharmacy, Casablanca, Morocco, N°02/19. A written informed consent was obtained from all participants before entering the study
